# Rabin's paradox for health outcomes

**DOI:** 10.1002/hec.3918

**Published:** 2019-06-19

**Authors:** Stefan A. Lipman, Arthur E. Attema

**Affiliations:** ^1^ Erasmus School of Health Policy and Management Erasmus University Rotterdam Rotterdam The Netherlands

**Keywords:** expected utility, loss aversion, Rabin's paradox, reference dependence, risk aversion

## Abstract

Many health economic studies assume expected utility maximisation, with typically a concave utility function to capture risk aversion. Given these assumptions, Rabin's paradox (RP) involves preferences over mixed gambles yielding moderate outcomes, where turning down such gambles imply absurd levels of risk aversion. Although RP is considered a classic critique of expected utility, no paper has as of yet fully tested its preferences within individuals. In an experiment we report a direct test of RP in the health domain, which was previously only considered in the economic literature, showing it may have pervasive implications here too. Our paper supports the shift towards alternative, empirically valid models, such as prospect theory, also in the health domain. These alternative models are able to accommodate Rabin's paradox by allowing reference‐dependence and loss aversion.

## 1 INTRODUCTION

Risk is central in health economics and is for instance covered in literature on health insurance (e.g., Arrow, 1963, Kairies‐Schwarz, Kokot, Vomhof, & Weßling, 2017), health state valuations (e.g., Pliskin, Shepard, & Weinstein, 1980; Torrance, 1976), health‐related behaviour change (e.g., Anderson & Mellor, 2008), and patient preferences (e.g., Galizzi, Miraldo, & Stavropoulou, 2016; Seston, Ashcroft, & Griffiths, 2007). Generally, individuals dislike risk, that is, variance in outcomes, and prefer certain options with the same expected value over risky options. This is referred to as risk aversion, a hallmark phenomenon in economics that has found its way into many health applications, such as Arrow's (1963) classic exposition on health insurance.

Risk aversion is typically modelled within the framework of expected utility (EU) theory with a concave utility function over wealth (Von Neumann & Morgenstern, 1944), often considered to be the normative and rational benchmark for decision making under risk (e.g., Harsanyi, 1955; Kahneman & Tversky, 1979; Wakker, 2010). However, the descriptive validity of EU, that is, its applicability to understand or describe how individuals actually decide, has been questioned for decades (for a review, see, Starmer, 2000). For example, several paradoxes have been presented in the economic literature that violated EU, such as Allais' (1953) paradox and Rabin's paradox (RP, Rabin & Thaler, 2001, Rabin, 2000). As an illustration of the latter: consider an agent who turns down a 50/50 gamble of gaining 11$ or losing 10$ at all wealth levels. Rabin (2000) showed how under EU with concave utility this agent should turn any gamble with a 50/50 loss of 100$, even when the agent could gain millions!

This thought experiment has become a classic criticism of EU as a descriptive theory of risk aversion. Although EU can capture risk aversion by assuming a concave utility function over wealth, this utility function should be extremely concave to capture turning down 50/50 gambles over such small stakes (e.g., gaining 11$ or losing 10$), which leads to absurd predictions for larger stakes. Given that most classic work on risk aversion in economics assumed EU with concave utility, RP has generated much debate among economists. Whereas some authors argue that EU is simply not a plausible theory for risk aversion and propose a move towards reference‐dependent theories (e.g., Bleichrodt et al., 2017), others criticize the assumptions relevant to RP (Andersen et al., 2011; Harrison, Lau, Ross, & Swarthout, 2017). However, empirical evidence on the presence of RP is scarce, only a handful of empirical studies are available that tested RP, all in the economic domain. First, Cox, Sadiraj, Vogt, and Dasgupta (2013) observed RP preferences for financial outcomes in an incentive‐compatible study. Second, Bleichrodt et al. (2017) identified the causes of RP empirically, showing how a reference‐dependent model with loss aversion may explain RP (as already suggested by Rabin, 2000). A drawback of the first study, however, is that it involved highly unlikely outcomes (i.e., casino outcomes), and a drawback of the second study is that preferences for large stakes are not studied.

So far, both theoretical and empirical works have focused solely on Rabin's (2001, 2000) critique on EU in the monetary domain. There it has had vast implications, as citation scores show. It is well known that preferences for health outcomes may differ from decisions for financial outcomes (even at a neurological level, see Suter, Pachur, Hertwig, Endestad, & Biele, 2015). Such differences have been observed for time preferences (e.g., Attema, Bleichrodt, L'Haridon, Peretti‐Watel, & Seror, 2018; Chapman, 1996), ambiguity preferences (e.g., Curley, Eraker, & Yates, 1984), and, most relevant to our purposes, risk preferences (e.g., Suter, Pachur, & Hertwig, 2016; Weber, Blais, & Betz, 2002). Whereas Allais' paradox has been tested with health outcomes (Oliver, 2003), no such work exists for RP. Therefore, in this paper we extend RP to the health domain.

## 2 NOTATION AND FORMAL DEFINITIONS

We first introduce notation and define RP for monetary outcomes (*x*,*y*). We consider agents as modelled in EU (Von Neumann & Morgenstern, 1944), who face gambles of the form *x*_0.5_*y*, that is, *x* results with probability 0.5 and *y* otherwise. Preferences are denoted by the usual ≻, ≽ , and ∼, representing strict preference, weak preference, and indifference, respectively. Under EU, gambles are evaluated linearly in probabilities; that is *x*_0.5_*y* is evaluated by: 0.5 *U*(*x*)+0.5 *U*(*y*), where often *U*(·) is assumed to be a strictly increasing and concave utility function over final wealth (which is equal to initial wealth *I*_*w*_ with the outcome of the gamble incorporated). This concavity of *U*(·) is assumed to reflect risk aversion, which is defined as preferring a gamble's expected value with certainty over the actual gamble (Wakker, 2010).

The classic RP thought‐experiment starts with the assumption that an agent turns down a gamble *x*_0.5_*y* at all levels of initial wealth, that is, always prefers staying at *I*_*w*_ over the gamble for some *x* and *y*. For example, assume *x* = +11 and *y* =  − 10, and consider someone who always turns down (+11*$*_0.5_ − 10*$*).By means of a calibration process, Rabin (2000) showed that this person should also turn down gambles with extremely large expected value. To illustrate this calibration process, assume we observe such risk aversion for all *I*_*w*_ and utility over final wealth remains concave. Turning down (+11*$*_0.5_ − 10*$*) at all wealth levels implies that over each length of 21 dollars *U*^′^ drops by a factor of 10/11 (see Wakker, 2010). Such geometric decay is highly unlikely, as it implies that the marginal utility of each additional dollar diminishes expeditiously: take for example the decay of *U*^′^ on an interval of 4200$, which will be ${\left(\frac{10}{11}\right)}^{200}=5.26*10^{-9}$ (see the supporting information for more detail). However, these conclusions only hold if gambles such as +11*$*_0.5_ − 10*$* are indeed turned down at all wealth levels (or at least at many wealth levels^1^). Often, this empirical assumption is justified by observing that many agents will (ceteris paribus) reject such gambles at many (if not all) wealth levels, which led Rabin (2000) to assume that this gamble will also be turned down by a single agent at many (if not all) wealth levels.

Now, we extend RP to health outcomes ( H), which are quantifiable and real‐valued (e.g., hours of life). We consider agents as modelled by EU in two cases: (a) individual decisions—that is, agents deciding about their own health, and (b) societal decisions—that is, agents deciding as societal decision makers for population health.^2^ In both cases, $U\left(H\right)\;$is a strictly increasing and concave utility function over final *health*. For individual decisions, initial health *I*_*h*_ denotes an agent's life expectancy before a choice is considered, and for societal decisions *I*_*h*_ denotes the societal decision maker's judgement of society's initial health. In both cases final health is obtained by adding to *I*_*h*_ (gains) or subtracting from *I*_*h*_ (losses) the relevant health outcomes in gambles, that is, $\;L,\ell ,I_{h},ℊ,\text{and}\;G\in H$ (see Table 1 for details on outcomes). We let $ℊ\;\left(G\right)$ represent a moderate (large) health gain compared to initial health *I*_*h*_, and we let $\ell \;\left(L\right)$ denote a moderate (large) health loss compared to *I*_*h*_. As in the canonical example by Rabin and Thaler (2001), we test RP by setting *ℊ* = +11 and *ℓ* =  − 10 (e.g., +11 or − 10 hr of life). Like Rabin (2000) for monetary outcomes, we assume that if *ℊ*_0.5_*ℓ* is turned down by agents with many different levels of *I*_*h*_, this implies that such gambles are also turned down by one individual at many life expectancies (for individual decision) and for many society's initial health levels (for societal outcomes).^3^ Under these assumptions (according to Rabin's (2000) calibration theorem), if we replace gamble *ℊ*_0.5_*ℓ* with $G_{0.5}L$, with L=−100, this person should turn down gambles for any G (up to G=∞). Given the difficulties with grasping infinity, we elicit RP with G=10,000.

**Table 1:** Scenarios for Rabin paradox (RP) gamble‐pairs for individual and societal outcomes

Gamble‐pair	Scenario	Outcome
	*Individual*	
RP1	Imagine that it is possible to take a gamble that affects your remaining lifetime (e.g., living until 87). The outcome is added or deducted from your lifetime.	Hours
RP2	Imagine that you are 75 and will live with slight mobility problems (not able to walk more than 3 km). You can gamble to change your lifetime (longer or shorter).	Hours
RP3	Imagine you are 75 and will live until 85 with light back pain (e.g., treatable with mild painkillers). You can gamble to change your life time.	Hours
	*Societal*	
RP4	Imagine a chronic disease, which leads to considerable losses in quality and length of life. Normally this disease affects about 300,000 people in the Netherlands (e.g., cancer). A risky drug is developed, which may either increase the amount of cases or decrease the amount of cases.	Cases averted/extra cases
RP5	Imagine an outbreak of a fatal disease occurred. The disease will lead to considerable lives lost. You are considering to take a gamble, in which either 11 lives are saved or 10 additional lives are lost.	Casualties saved/extra casualties
RP6	Imagine you have the chance to obtain extra healthy life years for society, be means of an easy to implement, costless, 3 medical procedure. As a reminder: you do not know to whom these life years will be distributed. The procedure also has a chance of resulting in a reduction of healthy life years for society.	Life years

*Note*. Each gamble‐pair had the following forms, with numbers referring to different health outcomes depending on the pair: (a) Moderate Stake Gamble *ℊ*_*p*_*ℓ*: (+11, 0.5, −10) and (b) Calibrated Gamble $G_{p}L$: (+10.000, 0.5, −100)

We define RP as the following combination of preferences: *I*_*h*_ ≻ *ℊ*_0.5_*ℓ* and $I_{h}≺G_{0.5}L$, which constitutes a violation of EU with concave utility.^4^ Whenever subjects turn down (accept) both gambles (i.e., *I*_*h*_ ≻ (≺) *ℊ*_0.5_*ℓ* & $I_{h}≻\left(≺\right)\;G_{0.5}L$), we will say that they do not violate EU.

## 3 METHOD

### 3.1 Sample

*N* = 201 subjects were recruited by means of the Erasmus Research Participation System. All subjects were Business Administration students and were rewarded course credits for participation. The mean age of our sample was 20.29 (*SD* = 1.36) and 34% of our sample was female.

### 3.2 Procedure and design

This experiment was part of a larger study on preferences for health outcomes and was completed using Qualtrics Survey Software. Each subject completed all six RP gamble‐pairs, which each consisted of a moderate stake gamble (*ℊ*_0.5_*ℓ*) and a calibrated large stake gamble ( $G_{0.5}L$). The RP gamble‐pairs were grouped in two counterbalanced blocks (completed within‐subjects): three individual gamble‐pairs and three societal gamble‐pairs (presented in random order).

### 3.3 Stimuli

The exact scenarios for all six gamble‐pairs can be found in Table 1, and instructions are reprinted in the supporting information. In accordance with Bleichrodt et al. (2017) we only asked subjects if they would accept this gamble, to which they could respond “Yes” or “No”.

### 3.4 Additional measures

We collected demographic information on age, gender, body‐mass index, subjective health (0–100 scale from worst to best imaginable health), and happiness (1–10 scale from completely dissatisfied to completely satisfied with life as a whole).

## 4 RESULTS

As can be seen from Table 2, for all items a small majority of the sample rejects the gambles for moderate stakes, and a large majority generally accepts calibrated gambles. These proportions are all significantly larger than 50% (*χ*^2^'*s* (1, *N* = 201)6.81, *p's* *s* < .009) for all items but RP1 (*χ*^2^(1, *N* = 201) = 1.44, *p* = .23). Next, for each RP gamble‐pair we determined how many subjects showed RP preferences (see Table 2). Out of all four possible preference patterns within gamble‐pairs, RP preferences occurred most frequently (43–64%). However, a substantial part of the sample showed preference combinations consistent with EU by rejecting or accepting both gambles (individual: 13% and 39%, societal: 15% and 23%). Of all choices consistent with RP preferences a larger share (358 out of 632, i.e., 56%) occurred for societal outcomes (*χ*^2^(1, *N* = 632) = 11.16, *p* < .001). Inversely, the proportion of our samples' choices satisfying EU was smaller (227 out of 541, i.e., 42%)^5^ for societal outcomes *χ*^2^(1, *N* = 541) = 13.99, *p* < .001). We also qualified these results with mixed logistic regression (see the supporting information), which suggested that RP preferences were more frequent for societal outcomes after controlling for the demographics collected (as described in Section 3.4).

**Table 2:** RP gamble‐pairs with number of acceptances (acc.) vs. rejections (rej.) for moderate stake gambles (MSG, in columns) and calibrated gambles (in rows), with row and column totals (tot.)

Individual setting		RP1‐MSG ℊ_0.5_ℓ			RP2‐MSG ℊ_0.5_ℓ			RP3‐MSG ℊ_0.5_ℓ		
Calibrated Gambles		Rej.	Acc.	(Tot.)	Rej.	Acc.	(Tot.)	Rej.	Acc.	(Tot.)
RP1‐RP3 $G_{0.5}L$	**Rej.**	15	3	(18)	35	8	(43)	26	4	(30)
	**Acc.**	94^+^	89	(183)*	87^+^	71	(158)*	93^+^	78	(181)*
	**(Tot.)**	(109)	(92)		(123)*	(79)		(119)*	(82)	

Societal setting		RP4‐MSG *ℊ*_0.5_*ℓ*			RP5‐MSG *ℊ*_0.5_*ℓ*			RP6‐MSG *ℊ*_0.5_*ℓ*		
Calibrated Gambles		Rej.	Acc.	(Tot.)	Rej.	Acc.	(Tot.)	Rej.	Acc.	(Tot.)
RP4‐RP6 $G_{0.5}L$	**Rej**	25	8	(33)	14	3	(17)	49	7	(58)
	**Acc.**	119^+^	49	(168)*	127^+^	57	(184)	112^+^	33	(145)*
	**(Tot.)**	(144)*	(57)			(141)*	(60)		(161)*	(40)

Note. ^a^RP preferences are signified by ^+^.

* The total proportion is significantly larger than 50%, by a pairwise *χ*^2^‐test with p< 0.05.

Next, we explored to what extent RP preferences were stable within‐subjects, by calculating what proportion of our sample showed this combination of preferences across gamble‐pairs. As can be seen from Table 3, overall RP preferences were observed frequently, with the percentages of those showing RP preferences for all three gamble‐pairs being near equal for individual and societal outcomes. When considering the stability of these preferences between domains, it appeared that many individuals that had no RP preferences for individual outcomes did show RP preferences for societal outcomes. A series of analyses in the supporting information shows that these preferences were more consistent and stable than would be expected if they were generated by a population satisfying EU or being completely indifferent (i.e., noise). Furthermore, RP preferences were more consistent than would have been expected if all choices were made independently across all gamble‐pairs.

**Table 3:** Frequency (N) and proportion (%) of RP preferences counts (C) within‐subjects

N (%)		Societal				
		C = 0	C = 1	C = 2	C = 3	Total individual
Individual	C = 0	19 (9%)	21 (10%)	22 (11%)	22 (11%)	84 (42%)
	C = 1	2 (1%)	6 (3%)	9 (4%)	8 (4%)	25 (12%)
	C = 2	4 (2%)	5 (2%)	9 (4%)	9 (4%)	27 (13%)
	C = 3	8 (4%)	12 (6%)	18 (9%)	27 (13%)	65 (32%)
Total societal		33 (16%)	44 (22%)	58 (29%)	66 (33%)	

## 5 DISCUSSION

The goal of this study was to supplement the empirical literature on RP, by extending this classic critique of EU to the health domain. We replicate RP for health; that is, we observe risk aversion for gambles over moderate health stakes, which implausibly (and incorrectly for a majority of our sample) suggests that calibrated large stake gambles should also be turned down according to EU. These findings are in accordance with the two other empirical studies testing RP preferences in the monetary domain (Bleichrodt et al., 2017; Cox et al., 2013). Several different hypothetical health outcomes and contexts were used, where RP preferences were more pronounced for societal outcomes. To our knowledge, our study is one of the first finding risk aversion for moderate individual health outcomes,^6^ with another example being Breyer and Fuchs (1982) who consider gambles over days with a 2‐hr headache. Risk aversion for larger individual health outcomes, for example, in the range of 0.5 to 20 years of life, is observed frequently (e.g., Galizzi, Miraldo, Stavropoulou, & van der Pol, 2016, Attema, Brouwer, & L'Haridon, 2013, Attema, Brouwer, L'Haridon, & Pinto, 2016, Van Der Pol & Ruggeri, 2008, Oliver, 2018), albeit these studies used a different methodology (i.e., certainty equivalences). For societal outcomes, studies have, for example, found risk aversion for life years (Eraker & Sox, 1981) or lives (Kemel & Paraschiv, 2018).

However, a substantial part (30–52%) of our sample did not violate EU by accepting or rejecting both gambles, which is similar to that observed in the only direct test of RP in the economic literature (Cox et al., 2013). A direct comparison to Bleichrodt et al. (2017) is not possible, as they only tested risk aversion for small stakes, but the proportion of their sample that accepts small stake gambles is lower compared to our sample. A surprising and unique result of our study is that a small set of the sample rejects both gambles, and the strong concavity these preference imply could be considered absurd (Wakker, 2010). Whereas earlier work on RP focused on criticising the assumptions generating RP (e.g., Andersen et al., 2011; Harrison et al., 2017), or explaining its paradoxical nature (e.g., via reference‐dependence; Bleichrodt et al., 2017), our work suggests that for a nonnegligible group of individuals no paradox may exist to begin with. Nonetheless, turning down the opportunity of gaining over a year of life or saving 10,000 lives when risking moderate losses in lifetime or human lives seems difficult to justify. Whereas loss aversion may explain RP preferences, as Rabin (2000) suggested and Bleichrodt et al. (2017) established, it is straightforward to demonstrate that to explain rejecting both gambles loss aversion would need to be extreme. Hence, we offer two explanations for these preferences not related to risk aversion. First, especially relevant to individual outcomes, some individuals may not be willing to live any longer in the reduced health states (in scenarios 2 and 3). Such preferences are observed frequently for health states more severe than those under consideration here (i.e., maximum endurable time, Sutherland, Llewellyn‐Thomas, Boyd, & Till, 1982). A second explanation is that one may prefer not to take any gamble at all, out of the well‐known preference for inaction over action when risking adverse outcomes (i.e., omission bias, Spranca, Minsk, & Baron, 1991).

Some additional methodological limitations deserve mentioning. First, this study was not specifically designed to test the validity of the assumptions present in Rabin's (2000) calibration theorem. Given that some of these have been challenged in the economic domain (e.g., Andersen et al., 2011; Harrison et al., 2017), this provides opportunities for future work. For example, it could be determined if risk aversion indeed holds for many (if not all) levels of initial health, either by testing this for a single individual at many (hypothetical) ages or by comparing risk aversion between individuals with different ages, that are otherwise similar. Second, this study used a relatively small, homogeneous, convenience sample, which may limit its external validity. Nonetheless, it is common to start a new experiment in convenience samples and extend it afterwards to representative samples. Third, our study relied on hypothetical scenarios without real incentives. Although the importance of incentive‐compatibility for behavioural experiments in health has often been stressed (e.g., Galizzi & Wiesen, 2018), our goal of offering calibrated gambles in terms of health made such a procedure impossible. Furthermore, some evidence exists in the economic domain suggesting that risk preferences are not qualitatively different between hypothetical and incentive‐compatible gambles, although they may be more variable in the former (for reviews, see: Camerer & Hogarth, 1999, Hertwig & Ortmann, 2001). Finally, our definition of RP and method only allowed for strict preferences, whereas the small differences in rates of acceptance and rejection for individual gamble‐pairs suggest that part of our sample may actually have been indifferent for moderate stakes. However, if this was the case we would have observed less within‐subjects stability and importantly such indifferences would still yield RP, as indifference for moderate stake gambles still implies risk aversion, and thus strong concavity under EU (see Online Supplements).

## 6 CONCLUSION

This study has shown that the paradox proposed by Rabin (2000) is also relevant to health outcomes. Given its large impact in economics, its implications for health deserve further study. It poses a challenge to earlier work in health economics which described risk aversion by means of EU with concave utility over health outcomes, as this would lead to implausible conclusions for the large stakes often present in the health domain—this is the main message of Rabin (2000). This appears to hold especially for models describing societal decision‐making. Fortunately, by modelling preferences (over health outcomes) within a reference‐dependent framework such as prospect theory (Tversky & Kahneman, 1992), RP can be easily resolved. The increasing attention for such reference‐dependent frameworks in health economics (Abellan‐Perpiñan, Bleichrodt, & Pinto‐Prades, 2009; Attema et al., 2013; Lipman, Brouwer, & Attema, 2019; Pinto‐Prades & Abellan‐Perpiñan, 2012) seeking more accurate descriptive theories is supported by our findings. Although these reference‐dependent theories may be general enough to capture the strong risk aversion demonstrated by a small part of our sample, further investigation to understand how we decide about health under risk is clearly still needed.

## CONFLICT OF INTEREST

None.

## FUNDING SOURCES

None.

## Supporting information


Figure A1:
Example item for individual perspective (left panel: RP3 – MSG) and societal perspective (right panel: RP6 – MSG)
**Table B1:** Ranges of 12,000 for each unit of H under consideration in RP gamble‐pairs with real‐life examples of comparable magnitude
**Table C1:** Proportion of subjects divided by their number (C) of acceptances for gambles within each context. C = 0 implies rejection throughout, and C = 3 implies acceptance throughout
**Table D1:** Results of logistic mixed effects regression predicting the occurrence of RP preferences
**Table D2:** Frequency (N) and proportion (%) of RP preferences counts (C) within‐subjects, if all agents satisfy EU
**Table D3:** Frequency (N) and proportion (%) of RP preferences counts (C) within‐subjects, if all agents act completely randomly
**Table D4:** Frequency (N) and proportion (%) of RP preferences counts (C) within‐subjects, if all agents are indifferent for moderate stakes (with empirical distribution informing large stakes)
**Table D5:** Frequency (N) and proportion (%) of RP preferences counts (C) within‐subjects, if all agents are indifferent for moderate stakes (with empirical distribution informing large stakes)
**Table D6:** Frequency (N) and proportion (%) of RP preferences counts (C) within‐subjects, if all agents preferences informed by the empirical distribution (drawn independently)

## ENDNOTES

1 Much of the discussion surrounding RP has focused on this assumption, with its validity being questioned for example by Andersen et al. (2011) and Harrison et al. (2017). Rabin (2000) showed that gambles need not be turned down at all wealth levels, and Wakker (2010) discusses how gambles only need to be turned down over relatively small domains of initial wealth to produce absurd concavity under EU.

2 See the supporting information for more detail on the social welfare function.

3 Obviously, for health outcomes there is less to no evidence that such preferences hold for many individuals at many levels of initial health. In fact, some authors have suggested that utility might be kinked around individuals' subjective life expectancy; that is, such expectations about length of life are a reference point (van Nooten & Brouwer, 2004; Van Nooten, Koolman, & Brouwer, 2009). However, the focus in this paper is to extend RP *preferences* to health, and hence, we will not extensively test or discuss the *assumptions* that generate the paradox. Furthermore, although such kinked preferences around subjective life expectancy may invalidate the assumptions necessary to generate RP, they increase the need to consider reference‐dependent models for decisions about health. The limited evidence that we obtained to sustain Rabin's (2000) empirical assumptions is discussed in Online Supplements.

4 The definitions used here rely on strict preference (as our experiment only involves direct choices), but as shown in Appendix C the following preferences also constitute RP: *I*_*h*_∼*ℊ*_0.5_*ℓ* and $I_{h}≼G_{0.5}L$

5 The remaining 2% of all choices over gamble‐pairs consisted of accepting the moderate stake gamble, but turning down the calibrated gamble. Such preferences occurred for a negligible part of the sample and are not captured by RP preferences or EU. We will not discuss these counter‐intuitive preferences in more detail.

6 We do not refer to our stimuli as small stake gambles, as we object to labelling any health loss as small, especially when our gambles concern human lives.
