# Supplementary material for: Rabin's paradox for health outcomes
Source: Health Econ. 2019 Jun 19;28(8):1064–71. doi: 10.1002/hec.3918 (PMC6771755; doi:10.1002/hec.3918)
Supplement: Supplementary file 1 — Figure A1: Example item for individual perspective (left panel: RP3 – MSG) and societal perspective (right panel: RP6 – MSG) Table B1: Ranges of 12,000 for each unit of H under consideration in RP gamble‐pairs with real‐life examples of comparable magnitude Table C1: Proportion of subjects divided by their number (C) of acceptances for gambles within each context. C = 0 implies rejection throughout, and C = 3 implies acceptance throughout Table D1: Results of logistic mixed effects regression predicting the occurrence of RP preferences Table D2: Frequency (N) and proportion (%) of RP preferences counts (C) within‐subjects, if all agents satisfy EU Table D3: Frequency (N) and proportion (%) of RP preferences counts (C) within‐subjects, if all agents act completely randomly Table D4: Frequency (N) and proportion (%) of RP preferences counts (C) within‐subjects, if all agents are indifferent for moderate stakes (with empirical distribution informing large stakes) Table D5: Frequency (N) and proportion (%) of RP preferences counts (C) within‐subjects, if all agents are indifferent for moderate stakes (with empirical distribution informing large stakes) Table D6: Frequency (N) and proportion (%) of RP preferences counts (C) within‐subjects, if all agents preferences informed by the empirical distribution (drawn independently) [file HEC-28-1064-s001.docx]

**Online Supplements Rabin’s Paradox (RP) for Health Outcomes**

This online supplement file contains a total of four appendices that provide additional information on the gambles (appendix A), additional theoretical background describing the role of initial health (appendix B), discuss the definition of RP when subjects are indifferent for moderate stake gambles (appendix C) and present additional analyses and results with regard to the effect of demographic variables and stability of RP preferences across individual and societal outcomes (appendix D). Analysis scripts and data are available from the corresponding author on request.

**Appendix A: Instructions used for Rabin Paradox Gamble-Pairs**

**1. General Instructions**

In this part of the questionnaire we will offer you different gambles, in which you can gain or lose health outcomes. We ask you whether you would play this gamble. You can answer either positively (‘Yes, I would’) or negatively (‘No, I would not’). All gambles involve health outcomes, and we ask you to take different perspectives throughout this short survey. Furthermore, each gamble has the same chance of generating success or failure, namely 50%. The probability of success is not affected by any personal characteristics, and can be viewed as the result of flipping a single coin.

**2. Individual perspective (gamble-pairs: RP1, RP2 and RP3)**

In this part of the questionnaire, we ask you to imagine that the outcomes of the gamble will affect you personally. In other words, the health gains or losses that result from the gamble will affect your life time and/or quality of life. For each gamble, it will be clarified when the outcomes occur. Your task is to indicate if you would play this gamble, given that the outcomes affect your personal situation.

**3. Societal perspective (gamble-pairs: RP4, RP5, and RP6)**

For this part of the questionnaire, we ask you to take a societal perspective. In other words, you are to decide like a policy maker in health care, who cares for the outcomes of everyone in society together. Imagine that you do not know exactly how the outcomes will be distributed, nor do you know your own position in society.

*
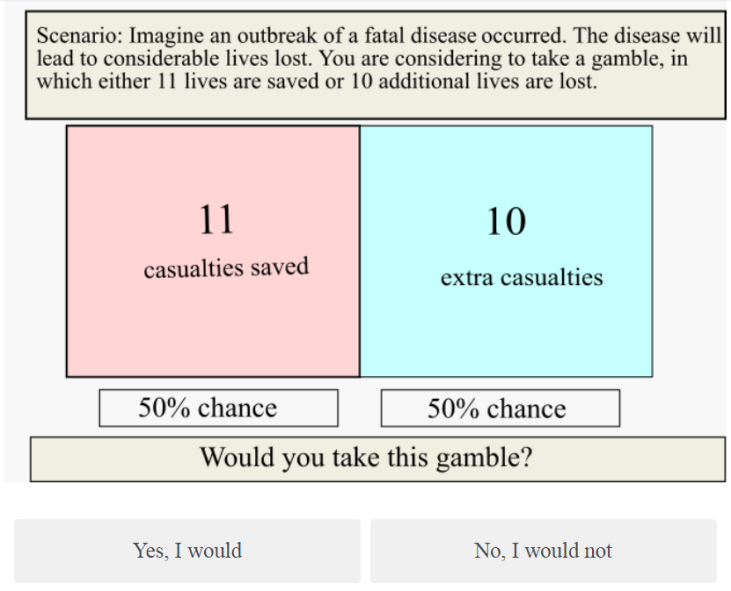
Figure A1: Example item for individual perspective (left panel: RP3 – MSG) and societal perspective (right panel: RP6 – MSG)*

*
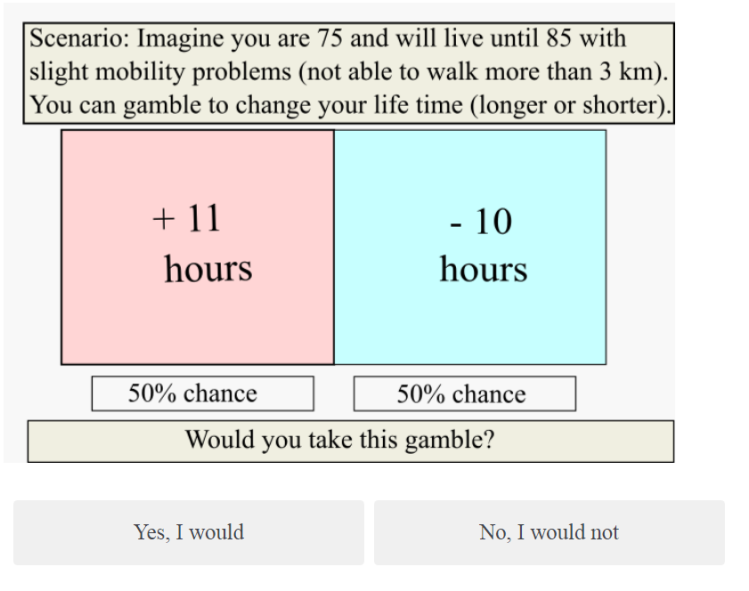
*

**Appendix B. Formalizing the role of initial wealth and health**

Wakker ([2010](#_ENREF_43)) provided a formalization of the role of initial wealth in Rabin’s ([2000](#_ENREF_31)) critique on EU based on calibration of moderate stake gambles. This formalization is instrumental to understand how plausible risk aversion over small gambles leads to implausible risk aversion over calibrated gambles – which could be accommodated by reference-dependence. Moreover, by extending this framework to health outcomes, we are able to allude to the plausibility of constant risk aversion over ‘common’ levels of $\mathcal{H.}$ Importantly, health outcomes, as opposed to monetary amounts, have several qualities that may hamper the generality of RP. We try to allude to these briefly in this Appendix. Throughout, we will use the notation defined in Section 2, and add to it where necessary.

EU is reference-independent, which for health outcomes indicates that outcomes are evaluated over final health$F_{h}\mathcal{\in H.}$ When considering gambles, such as the moderate stake gamble$\mathcal{g}_{p}\mathcal{l}$, the possible corresponding levels of $F_{h}$are: $I_{h}+\mathcal{g}$ or $I_{h}- \mathcal{l}$, signifying that outcomes designate changes from $I_{h}$. A crucial assumption of EU is that preferences are not affected by adding some amount to$I_{h}$and subtracting it from all outcomes in a gamble, as this does not affect final health. That is, EU over health outcomes $(\mathcal{H})$ holds if preferences over these outcomes can be represented by $U\left( \mathcal{H} \right).$ Preferences maximize the expectation of $U\left( \mathcal{H} \right)$ over final health, where $I_{h}$ is typically not denoted, as it is assumed constant to the agent and is usually unknown. In economics, this rescaling has been standard for decades ([Wakker, 2010](#_ENREF_43)).

The crucial assumption to obtain RP is risk aversion for common levels of $I_{h}$, which Wakker ([2010](#_ENREF_43)) refers to as Rabin’s empirical assumption (REA). That is, if ${0 \succ\mathcal{g}}_{0.5}\mathcal{l}$ at $I_{h}$, then for some ‘common’ range of possible levels of $I_{h}\mathcal{\in H}$, this preference should remain constant. As in Wakker ([2010](#_ENREF_43)), we are not able to completely formalize what ‘common levels for $I_{h}$’ refer to, but any range of 12,000 of such ‘common levels’ for the health outcomes under consideration (see Table B1) suffices for our definition of RP. In our experiment, we observed such preferences for $\mathcal{g}=11, p=\frac{1}{2},$and $\mathcal{l= -}11$. Table B1 suggests that such possible ranges appear to be conceivable and realistic for health outcomes and may occur in practice (e.g. for individual outcomes we have a difference of 1.4 year in life expectancy). By extending this assumption of constant risk aversion to health, i.e. for many ‘common levels of $I_{h}$’ we thus assume that: a) individuals with different ‘common levels of $I_{h}$’ will turn down gambles^[[1]](#footnote-1)^ of the form$\mathcal{g}_{0.5}\mathcal{l}$, at least on a range of up to 12,000 $\in\mathcal{H}$, b) as in Rabin (2000) we assume that if this between-subject risk aversion is likely for ‘common levels of $I_{h}$’, it shall also hold for a single subject at different levels of $I_{h}$, again at least on a range of 12,000$\in\mathcal{H}$.

**Table B1:** Ranges of 12,000 for each unit of $\mathcal{H}$ under consideration in RP gamble-pairs with real-life examples of comparable magnitude

| Gamble-pair | Ranges of 12,000 under consideration |
| --- | --- |
| Individual | |
| RP1-3 | Changes in life duration of 1.4 life year (12,000 hours) |
| Societal | |
| RP4 | 12,000 disease cases |
| RP5 | 12,000 casualties for an unspecified disease |
| RP6 | 12,000 healthy life years |

Under these assumptions, as Wakker ([2010](#_ENREF_43)) concludes in his Chapter 8.6, if EU in terms of final health holds, this implies that for many levels $I_{h}$and many non-zero outcomes $x\in\mathcal{H}$ we should have:

$$x\succ\left( 0.5: x+11, 0.5, x-10 \right).$$

If we, as Rabin ([2000](#_ENREF_31)) assumed, take $U\left( \mathcal{H} \right)$ to be strictly increasing and concave, we have:

$U\left( x+11 \right)-U\left( x \right)<U\left( x \right)-U(x-10)$, signifying that the marginal utility on the interval [x, x+11] is smaller than on the domain [x-10, x]. This implies that on the domain [x-10, x+11] of $\mathcal{H}$ of length 21, $U^{'}$ drops by a factor of at least 10/11 ([Wakker, 2010](#_ENREF_43)). Extending this to many levels $I_{h}$and many non-zero outcomes $x$, we can calibrate this decrease in marginal utility to larger domains, by taking multiples of length such as 42 $(({\frac{10}{11})}^{2}=0.83)$, 63,$(({\frac{10}{11})}^{3}=0.75)$, 210, $(({\frac{10}{11})}^{10}=0.39)$, 2100 $(({\frac{10}{11})}^{100}=0.00073)$ and 4200 $(({\frac{10}{11})}^{200}=5.26*{10}^{-9})$. Wakker ([2010](#_ENREF_43)) postulates that such geometric decay is absurd. This implausible result can be accommodated by taking into account reference-dependence (see [Bleichrodt et al., 2017](#_ENREF_9), [Wakker, 2010](#_ENREF_43)).

Additionally, given our aim to apply RP to both individual and societal health outcomes, we will allude to extending this theoretical framework based on Wakker ([2010](#_ENREF_43)) to a social decision-maker facing gambles over societal health outcomes. We assume that the agent is a social planner deciding from under a Rawlsian veil of ignorance; that is, the agent does not know to whom the outcomes in each gamble will accrue. As we do not allude to (unequal) distributions in our scenarios, for simplicity, we will assume that the societal decision maker aims to maximize health ([for a discussions of equity and maximizing health, see: Culyer and Wagstaff, 1993](#_ENREF_14)). Furthermore, we will assume monotonicity, i.e. the societal decision maker prefers allocations providing more health for society as a whole over those providing less. Gambles of the form of $\mathcal{g}_{0.5}\mathcal{l}$ indicate a gamble between gaining and losing a small amount of health for society. As in EU, preferences are defined over final population health $F_{hp}$, which is $I_{hp}$ (initial population health) with the outcomes of gambles incorporated, i.e. the social planners welfare function is reference-independent. Furthermore, we assume that our social planner is risk averse, which, as in EU, is exclusively modelled through the welfare function. When we consider societal outcomes we, thus, assume $W$ to be a concave function over final societal welfare.

**Appendix C: On indifference for moderate stakes**

The theoretical framework and design used in this study are based on strict preferences between rejecting or accepting moderate and large stake gambles. That is, if subjects were indifferent between accepting or rejecting some gamble, this is not captured by our data and does not fit the definitions for RP preferences or preferences that do not violate EU. Typically, indifference is defined as follows: $x \sim y \Longleftrightarrow x≽ y$and $x≼ y.$Hence, given the choice between $x$ and $y,$ an indifferent subject should be equally likely to pick either of these two outcomes. When we extend this to the stimuli in our study, where subjects were offered the choice to accept or reject gambles of the form $x_{0.5}y$, indifference would imply: $x_{0.5}y≽0$ (accept) and : $x_{0.5}y≼0$ (reject). It follows that truly indifferent subjects would be equally likely to accept or reject the gambles, and the results in Table 2 give some indication that this may have been the case for individual moderate stake gambles (RP1-RP3). It can be observed that only a slight majority rejected the gamble, which was not significant for RP1. Hence, at least when observing between-subjects, it could be concluded that indifference for moderate stake gambles was likely.

However, if subjects were truly indifferent, it should also hold that rates of acceptance and rejection are split equally *within-subjects*, i.e. a subject who is truly indifferent for any series of gambles should have accepted once or twice, and rejected the other gambles (with fewer exceptions at the extremes of rejecting all three or accepting all three gambles, for which the expected proportion is 12.5%). Table C1 shows these proportions for our sample. The large proportions of subjects showing consistent rates of acceptance or rejection throughout, lead to the conclusion that it is unlikely that the majority of the sample was truly indifferent between accepting and rejecting the moderate and large stake gambles in this experiment.

**Table C1:** Proportion of subjects divided by their number (C) of acceptances for gambles within each context. C=0 implies rejection throughout, while C=3 implies acceptance throughout

| Gamble | C=0 | C=1 | C=2 | C=3 |
| --- | --- | --- | --- | --- |
| Individual – moderate stakes | 63 (31%) | 40 (20%) | 85 (42%) | 13 (6%) |
| Individual – large stakes | 67 (33%) | 96 (48%) | 18 (9%) | 20 (10%) |
| Societal – moderate stakes | 36 (18%) | 36 (18%) | 105 (52%) | 24 (12%) |
| Societal – large stakes | 32 (16%) | 109 (54%) | 55 (27%) | 5 (2%) |

However, it is not possible to rule out that indifference may have occurred. Hence, the definitions of risk aversion, RP preferences, and non-violation of EU would not hold for some subjects. A slight modification of notation and definitions, nonetheless, shows that, for subjects who are in fact indifferent between accepting or rejecting moderate stake gambles, such indifference still implies risk aversion and thus strong concavity on a small domain (as elaborated on in Appendix B). If such preferences, as is assumed in REA, hold for all levels of $I_{h}$, or at least on a sufficient range (e.g. in the ranges in B1), indifference for moderate stakes would still imply RP if large stake gambles are accepted. We elaborate below.

Risk aversion is traditionally defined as preferring the expected value of a gamble to (playing) the gamble itself. Hence, it implies $x_{0.5}y\prec0.5*x+0.5*y$. On the other hand, risk neutrality implies that $x_{0.5}y\sim0.5*x+0.5*y$. In other words, a risk averse person always prefers the expectation over the gamble while a risk neutral person is indifferent between the expectation and the gamble. By extension, if we assume that $U\left( \mathcal{H} \right)$ is increasing (which uncontroversially implies that more health gives more utility), a risk neutral person will always have $x_{0.5}y\succ0,$ whenever $0.5*x+0.5*y>0,$ i.e. the gamble has positive expected value. Hence, indifference for moderate stake gambles $\mathcal{g}_{0.5}\mathcal{l}$with $\mathcal{g}=11$ and $\mathcal{l}$ =-10 violates risk neutrality as the expectation of the gamble is positive. Without any further assumptions with regard to the shape of $U\left( \mathcal{H} \right)$, other than that it is increasing, we can conclude that this person, who is indifferent for moderate stakes gambles, has a certainty equivalent that is smaller than the utility of the expected value (i.e. $U(0)$ > U($0.5*\mathcal{g}+0.5*\mathcal{l))}$. That is, indifference still implies risk aversion for moderate stakes. It is then straightforward to conclude that if EU with concave $U\left( \mathcal{H} \right)$ should hold, the analysis in Appendix B still holds, except that instead of $U\left( \mathcal{H} \right)$ dropping by a factor of at least 10/11, it drops by a factor of exactly 10/11, which leads to absurd geometric decay. Hence, indifference for moderate stake gambles implies RP, but the result is weaker.

Given that our definition of RP requires rejection, but indifference is a sufficient condition for RP, this shows that our conclusions are not only robust to this methodological limitation, but it is actually likely that the degree of RP is underestimated. More specifically, subjects that accept moderate stake gambles are currently in no case defined as showing RP preferences, while if they actually were indifferent they may still have violated EU if they accepted a calibrated gamble.

**Appendix D: Supplementary analyses and results**

D1: Fixed effects model for RP preferences and demographics

We supplement our descriptive findings by running a logistic mixed effects regression models (with the R package lmerTest) on the likelihood of subjects showing RP preferences. The model included subject random effects, and fixed effects for several demographics (as described under ‘Additional measures’ in Section 3 of the main text): a) age, b) sex, c) BMI, d) subjective health, and e) happiness. Additionally, we include a fixed effect for outcome domain (i.e. individual vs. societal). As can be seen from Table D1, these analyses indicate that only outcome domain is a significant predictor of RP preferences, with RP preferences being more likely in the societal domain.

**Table D1:** Results of logistic mixed effects regression predicting the occurrence of RP preferences

|  | *Estimate* | *SE* | *z* | *p* |
| --- | --- | --- | --- | --- |
| Constant | -1.37 | 1.07 | 1.283 | 0.20 |
| Age | 0.006 | 0.05 | 0.137 | 0.89 |
| Sex | 0.07 | 0.12 | 0.649 | 0.52 |
| BMI | < -0.001 | <0.001 | -0.125 | 0.90 |
| Subjective health | -0.002 | 0.013 | -0.33 | 0.74 |
| Happiness | 0.07 | 0.022 | 1.647 | 0.10 |
| Outcome domain | 0.56 | 0.030 | 4.82 | **<0.001** |

D2: Elaboration of results on within-subjects stability of RP preferences (Table 3).

Next, in this appendix we report the results of a series of analyses, in which we provide further evidence that the RP preferences observed in this paper a) are not generated by noise, and b) are correlated within scenarios. To that end, we will compare our empirically obtained distribution of RP preferences (Table 3) against a series of null-hypotheses, and test if our distribution is significantly different by means of $\chi^{2}$ tests.

1. Null hypothesis: all agents satisfy EU

If all agents satisfy EU, no RP preferences should occur, as these preferences by definition violate EU (see section 2 and Appendix B). Thus under this null hypothesis Table 3 should have shown the results presented in Table D2.

**Table D2:** Frequency (N) and proportion (%) of RP preferences counts (C) within-subjects, if all agents satisfy EU

| N (%) |  | Societal |  |  |  |  |
| --- | --- | --- | --- | --- | --- | --- |
|  |  | C = 0 | C = 1 | C = 2 | C = 3 | Total individual |
| Individual | C = 0 | 201 (100%) | 0 (0%) | 0 (0%) | 0 (0%) | 201 (100%) |
|  | C = 1 | 0 (0%) | 0 (0%) | 0 (0%) | 0 (0%) | 0 (0%) |
|  | C = 2 | 0 (0%) | 0 (0%) | 0 (0%) | 0 (0%) | 0 (0%) |
|  | C = 3 | 0 (0%) | 0 (0%) | 0 (0%) | 0 (0%) | 0 (0%) |
| Total societal | | 201 (100%) | 0 (0%) | 0 (0%) | 0 (0%) |  |

Obviously, performing a χ^2^–test for this null hypothesis is mathematically impossible, given that no RP preferences were expected at all. That is, by definition under this null hypothesis, when applying a χ^2^-testwe will find $\chi^{2}(df=15)= \infty, p=0$ for our observed results in Table 3.

1. Null-hypothesis: all agents act completely randomly or are completely indifferent

If all agent have random preferences the chance that they accept any of the moderate stake- or calibrated gambles is exactly 50%. This also holds if all agents are indifferent between rejecting and accepting both the moderate and large stake gambles. Given that RP preferences are only one out of four possible combinations of preferences (as seen in Table 2), the chance of showing RP preferences for a single gamble-pair is 25%. Under these assumptions, by probability calculus we can derive the results that Table 3 should have given for $n$*= 201),* as can be seen in Table D3.

**Table D3:** Frequency (N) and proportion (%) of RP preferences counts (C) within-subjects, if all agents act completely randomly

| N (%) |  | Societal |  |  |  |  |
| --- | --- | --- | --- | --- | --- | --- |
|  |  | C = 0 | C = 1 | C = 2 | C = 3 | Total individual |
| Individual | C = 0 | 36 (18%) | 36 (18%) | 12 (6%) | 1 (<1%) | 85 (42%) |
|  | C = 1 | 36 (18%) | 36 (18%) | 12 (6%) | 1 (<1%) | 85 (42%) |
|  | C = 2 | 12 (6%) | 12 (6%) | 4 (2%) | 0 (0%) | 28 (14%) |
|  | C = 3 | 1 (<1%) | 1 (<1%) | 0 (0%) | 0 (0%) | 2 (1%) |
| Total societal | | 85 (42%) | 85 (42%) | 28 (14%) | 2 (1%) |  |

For this null-hypothesis we observed $\chi^{2}(df=15)> 15000, p<0.001$, indicating that if subjects were acting randomly the results we observed in Table 3 were extremely unlikely.

1. Null-hypothesis: all agents are indifferent for moderate stakes gambles

Our design does not allow distinguishing between those that are indifferent between accepting and rejecting gambles or strictly prefer one of these options. If all subjects were indeed indifferent, they would be choosing at random for moderate stakes, and Table 2 gives some indication that this may have been the case (only a small majority rejects moderate stake gambles for individual gamble-pairs). Hence, for this null hypothesis, we assumed that all agents are indifferent for moderate stakes (i.e. 50% of accepting or rejecting), and let the probability of accepting or rejecting the large stake gambles depend on the observed frequencies in our sample. Under these assumptions we can derive the expected results for Table 3 for *n=201* analytically or by resampling from the empirical distribution (see Table D4, details and script available on request)*.*

**Table D4:** Frequency (N) and proportion (%) of RP preferences counts (C) within-subjects, if all agents are indifferent for moderate stakes (with empirical distribution informing large stakes).

| N (%) |  | Societal |  |  |  |  |
| --- | --- | --- | --- | --- | --- | --- |
|  |  | C = 0 | C = 1 | C = 2 | C = 3 | Total individual |
| Individual | C = 0 | 8 (4%) | 16 (8%) | 11 (5%) | 3 (1%) | 38 (19%) |
|  | C = 1 | 17 (8%) | 36 (18%) | 26 (13%) | 6 (3%) | 85 (42%) |
|  | C = 2 | 13 (6%) | 27 (13%) | 19 (9%) | 4 (2%) | 63 (31%) |
|  | C = 3 | 3 (1%) | 7 (3%) | 5 (2%) | 1 (<1%) | 16 (8%) |
| Total societal | | 31 (15%) | 86 (43%) | 61 (30%) | 14 (7%) |  |

For this null-hypothesis we observed $\chi^{2}(df=15)> 946.71, p<0.001$, indicating that if subjects were truly indifferent for moderate stakes the results we observed in Table 3 were extremely unlikely.

1. Null hypothesis: agents show preferences based on the empirical rates of acceptance and rejection, but these preferences are not correlated between gambles

For this null hypothesis, we assumed that choices on each gamble are drawn independently from the empirical distribution. Consider for example that for RP1 the chance of rejecting the gamble was 109/201 for moderate stakes and 18/201 for large stakes. Under these assumptions we can derive the expected results for Table 3 for *n=201* analytically or by resampling from the empirical distribution () (see Table D5, details and script available on request)*.* This allows us to compare the empirical distribution of RP preferences across individual and societal outcomes to a null hypothesis in which no correlation exists between any gambles.

**Table D5:** Frequency (N) and proportion (%) of RP preferences counts (C) within-subjects, if all agents are indifferent for moderate stakes (with empirical distribution informing large stakes).

| N (%) |  | Societal |  |  |  |  |
| --- | --- | --- | --- | --- | --- | --- |
|  |  | C = 0 | C = 1 | C = 2 | C = 3 | Total individual |
| Individual | C = 0 | 2 (1%) | 7 (3%) | 11 (5%) | 6 (2%) | 25 (12%) |
|  | C = 1 | 5 (2%) | 22 (11%) | 33 (16%) | 17 (8%) | 77 (38%) |
|  | C = 2 | 4 (2%) | 21 (10%) | 32 (16%) | 16 (8%) | 74 (37%) |
|  | C = 3 | 1 (<1%) | 7 (3%) | 10 (5%) | 5 (2%) | 23 (11%) |
| Total societal | | 13 (6%) | 57 (28%) | 85 (42%) | 43 (21%) |  |

For this null-hypothesis we observed $\chi^{2}(df=15)> 464.27, p<0.001$, indicating that if all gambles were truly accepted or rejected independently from each other, the results we observed in Table 3 were extremely unlikely.

1. Agents show RP preferences based on the empirical data, but these RP preferences are not correlated between gamble-pairs

For this final null hypothesis, instead of assuming that choices on all gambles are independent of each other, we assumed that each gamble-pair (i.e. a combination of moderate and large stake gamble) is drawn independently from the empirical distribution. That is, for RP1 the chance of showing RP preferences is 94/201, while for RP4 it is 119/201. This procedure allows us to compare the empirical distribution of RP preferences across both outcomes to a null hypothesis where no correlation exists between gamble-pairs for these two outcomes (see Table D6).

**Table D6:** Frequency (N) and proportion (%) of RP preferences counts (C) within-subjects, if all agents preferences informed by the empirical distribution (drawn independently).

| N (%) |  | Societal |  |  |  |  |
| --- | --- | --- | --- | --- | --- | --- |
|  |  | C = 0 | C = 1 | C = 2 | C = 3 | Total individual |
| Individual | C = 0 | 2 (1%) | 10 (3%) | 14 (7%) | 7 (3%) | 25 (12%) |
|  | C = 1 | 5 (2%) | 24 (17%) | 35 (16%) | 17 (8%) | 77 (38%) |
|  | C = 2 | 5 (2%) | 20 (10%) | 29 (14%) | 14 (8%) | 74 (37%) |
|  | C = 3 | 1 (<1%) | 6 (3%) | 8 (4%) | 4 (2%) | 23 (11%) |
| Total societal | | 13 (6%) | 60 (30%) | 85 (42%) | 43 (21%) |  |

For this null hypothesis we observed $\chi^{2}(df=15)> 441.14, p<0.001$, indicating that if gamble-pairs were truly completed independently across individual and societal tasks, the results we observed in Table 3 were extremely unlikely.

**Conclusion**

Collectively, these five analyses lead to the following conclusion. First, it is impossible that our results were generated by a sample completely consisting of subjects that satisfy EU. Second, it is highly unlikely that our results were generated by a sample consisting completely of subjects that acted randomly, either because of lack of involvement or due to being ‘truly’ indifferent. Third, our results are not likely to be generated by subjects that were indifferent for moderate stake gambles, as we find more subjects who consistently show RP preferences for all gamble-pairs, or consistently show RP preferences for societal gambles but not for individual gambles. Fourth, these two patterns (i.e. RP throughout and RP for societal but not individual outcomes) occur more frequently than would be expected if no correlation existed between choices on all gambles (Table D5) or between gamble-pairs (Table D6).

1. Our paper provides some evidence for this empirical claim, as even in our homogeneous sample subjects turning down the gamble will have varying expectations about their length of life (see for example Pentek et al., 2014), and will thus have different $I_{h}$. [↑](#footnote-ref-1)
